# Antioxidant and Antimicrobial Potentials of Seed Oil from *Carthamus tinctorius L.* in the Management of Skin Injuries

**DOI:** 10.1155/2020/4103418

**Published:** 2020-11-04

**Authors:** Ikram Khémiri, Badiaa Essghaier, Najla Sadfi-Zouaoui, Lotfi Bitri

**Affiliations:** ^1^Unité de Physiologie des Systèmes de Régulations et des Adaptations, Faculté des Sciences de Tunis, Université de Tunis El Manar, Campus Universitaire, 2092 Tunis, Tunisia; ^2^Laboratoire de Mycologie, Pathologies et Biomarqueurs, Faculté des Sciences de Tunis, Université de Tunis El Manar, Campus Universitaire, 2092 Tunis, Tunisia

## Abstract

Infection of skin injuries by pathogenic microbial strains is generally associated if not treated with a lasting wound bed oxidative stress status, a delay in healing process, and even wound chronicity with several human health complications. The aim of the current study was to explore the antioxidant and antimicrobial potentialities of safflower (*Carthamus tinctorius L.*) extracted oil from seeds by cold pressing which would be beneficial in the management of skin wounds. Antioxidant capacity of the oil was evaluated (scavenging ability against 1,1-diphenyl-2-picrylhydrazyl radical (DPPH) and 2,2′-azino-bis 3-ethylbenzothiazoline-6-sulfonic acid (ABTS), and ferric reducing antioxidant power (FRAP)). Total phenolic, total flavonoid, total carotenoid, and total chlorophyll contents were determined. Antimicrobial activities of safflower oil were tested against 10 skin pathogenic microorganisms: 4 bacterial strains (*Escherichia coli*, *Enterobacter cloacae*, *Staphylococcus aureus*, and *Streptococcus agalactiae*), 3 yeast species strains (*Candida albicans*, *Candida parapsilosis*, and *Candida sake*), and 3 fungi species *(Aspergillus niger*, *Penicillium digitatum*, and *Fusarium oxysporum*). A notable antioxidant capacity was demonstrated for the tested oil that exhibited moreover high antibacterial effects by both bacteriostatic and bactericidal pathways including lysozyme activity. An antifungal effect was further observed on the spore's germination. Safflower oil could be considered as a good natural alternative remedy in the management of skin wounds and their possible microbial infections.

## 1. Introduction

The skin is one of the most important organs in the human body in weight, surface, and function. Mainly, it ensures a physical protection from mechanical and chemical trauma added to an immunological defence against environmental microbiota. The loss of parts or all of the skin layers (epidermis and dermis) causes a breakdown of the protective function of this organ. The most common skin injuries are erosions, lacerations, punctures, surgical wounds, ulcers, and burns. Wound healing process main target is to restore the integrity of the skin barrier and thus preserve the body's health [1]. Despite the fact that acute skin injuries can heal spontaneously within a few days, it is mandatory in several cases to take care of the wounds in order to help the process along and avoid infections and complications which can range from minor self-limited infections [2] to life-threatening invasive clinical diseases [3–5].

Untreated skin wound infections have been reported to be detrimental to the healing process, inducing wound chronicity, morbidity, and even mortality [6–8]. This is of wide concern in public health, especially in geriatrics [9, 10]. Diabetic, pressure, and vascular ulcers are the major forms of chronic wounds [11, 12]. Due to peripheral vascular disease, reduced blood flow and thus reduced tissue nutrition and oxygenation can lead to increased vulnerability of the skin to pathogens from the surrounding microbiota [9, 12]. Poorly treated decubitus ulcers in bedridden or elderly patients can even cause sepsis, especially in nosocomial infections. This is due to the fact that older adults are more likely to develop infectious complications [13, 14].

Several studies have reported that the colonization of the wound bed with opportunistic skin pathogenic strains (bacteria, yeast, and fungi) is commonly associated with infections and a delay of wound closure [15–17]. Bacteria belonging to the genera *Staphylococcus*, *Enterococcus*, *Enterobacter*, *Escherichia*, and *Streptococcus* have been reported to be associated with skin infections in the case of postsurgical complications, certain burns (especially second- and third-degree burns), and diabetic foot ulcers and bedsores [18–22]. They have been related to produce biofilms which are sometimes persistent and hard to treat and eradicate [23–25]. Other studies have also shown the involvement of other microorganisms such as yeasts of the genus *Candida* and fungi of the genera *Aspergillus*, *Fusarium*, and *Penicillium* in the infection of skin wounds and the impairment of the wound healing process [16, 22, 26, 27].

Over the recent past decades, the widespread use and overconsumption of systemic and topical medicines have led to the emergence of drug-resistant microbial strains [28, 29] such as methicillin-resistant *Staphylococcus aureus* [14, 30–32] and multiresistant *Candida* species [33] which caused serious public health problems.

The cutaneous wound healing is a dynamic and definite organized process which can be divided into four major overlapping steps: hemostasis to stop local hemorrhage after blood capillary rupture, inflammation to fight contaminating microorganisms, angiogenesis and reepithelialization to close the wound, and finally maturation and remodeling of the scar. Since the early phases of the inflammatory process, the wound bed is invaded by inflammatory cells such as neutrophils followed by lymphocytes and monocytes. They release prooxidant mediators such as reactive oxygen species (ROS) like hydroxyl radical (OH^∗^-), hydroxyl peroxide (H_2_O_2_), and superoxide radical anion (O_2_^∗^-) and reactive nitrogen species (RNS) such as nitric oxide (NO) in sufficient amounts in order to create a molecular microenvironment beneficial to microbial cell lysis [3, 34–36]. After that, several antioxidants are released in the wound bed to counteract the prooxidant factors thanks to their free-radical scavenging properties and thus quench damage caused by oxidative stress which leads to disturbances in cell physiology (lipids, proteins, carbohydrates, and DNA) and even to necrosis. Insufficient amounts of endogenous antioxidants especially in elderly and/or immune-depressed patients with underlying pathologies could compromise the healing process and lead to wound chronicity and complications.

Various beneficial health effects of herbal extracts have been proven and used since antiquity and continue to be so nowadays. There is a worldwide growing interest of researchers to prospect new pharmaceutical approaches, mainly based on ethnopharmacological relevance bioactive compounds, that can be extracted from all parts of plants (flowers, fruits, leaves, stems, roots, and seeds) [37–39]. Several studies have reported the effectiveness of nontoxic plant extracts in wound healing [40–45].

Fixed vegetable oils extracted from seeds are natural extracts rich in several active biocompounds such as fatty acids, vitamins, and sterols [46]. The investigation of potential effects of fixed vegetable oils and their different components in the healing of skin wounds has attracted the interest of several researchers around the world [47–52].

Safflower (*Carthamus tinctorius L.*), a member of the *Asteraceae* family, is a thistle-like herbaceous annual plant. It can withstand arid or semiarid climates with seasonal rainfall. Its orange-colored flowers are rich in carthamin, an orange or red pigment used as a dye [53]. The seeds of apical capitulas harvested at the latest vegetative stage are rich in a slightly yellow colored edible oil. *Carthamus tinctorius L.* is one of the mostly used aromatic herbals for medicinal purposes, especially in traditional pharmacopeias. Various studies have been carried out on some parts of this plant such as leaf and flower extracts [54–56] and their biological effects.

The present work was designed to study the antioxidant and antimicrobial potentials, beneficial in the treatment of skin wounds, of the natural vegetable oil extracted by cold pressing of safflower (*Carthamus tinctorius L.*) seeds.

## 2. Materials and Methods

### 2.1. Safflower Crude Edible Seed Oil Extraction

Safflower (*Carthamus tinctorius L.*) seeds were harvested in June 2017 from flower heads of plants grown in the northwest of Tunisia. The seeds were then sieved manually in order to clean them from impurities and dust. The edible seed oil was extracted by first cold pressing, using a machine (SMIR, MUV2 65). This method does not use any chemical products during the extraction process and ensures the preservation of oil components. The extracted seed oil was then stored until use in anti-UV hermetic clean bottles as previously described [57].

### 2.2. Physicochemical Properties of Extracted Safflower Seed Oil

The tests were carried out according to the official methods of AOCS (American Oil Chemists' Society, International). Moisture ratio of the dried seeds was estimated according to ISO 665:2000. Saponification index was assessed according to the Norm ISO 3657:2013. Refractive index was determined with an Abbe refractometer with temperature adjustment at 20°C. Peroxide value was determined in meq O_2_/kg of oil according to NF T60-220:1998. Iodine value (g *I*_2_/100 g oil) and acid index (mg KOH/g oil) were calculated according to the AOAC official method 940.28, 2013, and NF ISO 660-1996, respectively. Density was estimated at 20° as mass/volume (g/cm^3^).

### 2.3. Determination of Safflower Oil Antioxidant Capacity

#### 2.3.1. Scavenging Ability against Free Radicals DPPH (1,1-Diphenyl-2-Picrylhydrazyl Radical) and ABTS (2,2′-Azinobis-3-Ethylbenzothiazoline-6-Sulfonate)

Scavenging activity of Safflower seed oil against DPPH and ABTS free radicals was evaluated as described [58]. 180 *μ*L of 0.1 mM DPPH solution was mixed with 20 *μ*L of safflower oil. The mixture was shaken vigorously then left to incubate at room temperature in the dark for 30 min. The absorbance was measured at 520 nm using a spectrophotometer (Thermo Fisher Scientific Multiskan Go).

In order to produce ABTS radical cation, 2.5 mM potassium persulphate was mixed with 7 mM ABTS solution (at pH 7.4) and left in the dark for 16 h. A dilution of the mixture was carried out until absorbance of 0.70 ± 0.02 at 734 nm was reached. 180 *μ*L of fresh ABTS diluted solution was added to 20 *μ*L of the dissolved oil sample. Absorbance (*A*) was measured 6 min after the mixing. The maximum absorbance of DPPH and ABTS of the sample was recorded as *A*_sample_ and of the blank control as *A*_blank_. The measurements were performed in triplicate. The results were expressed as Vit. C eq/g oil. Ascorbic acid was used as a standard. The percentage inhibition (%) of the free radical DPPH and ABTS was calculated as follows:

(1) $$\begin{array}{c} I\%=\left[\frac{A_{\text{blank}}-A_{\text{sample}}}{A_{\text{blank}}}\right]\times 100. \end{array}$$

#### 2.3.2. Ferric Reducing Antioxidant Power (FRAP)

The FRAP of the safflower seed oil was evaluated using the method of Benzie and Strain [59] with slight modifications [60]. The fresh FRAP reagent was prepared daily as required, by mixing 2.5 mL of 20 mM ferric chloride hexahydrate (FeCl_3_·6H_2_O) solution with 25 mL of acetate buffer 0.1 M (pH 3.6) (CH_3_COONa-3 H_2_O; CH_3_COOH) and 2.5 mL of 10 mM TPTZ (2,4,6-tri(2-pyridyl-s-triazine) solution in 40 mM hydrochloric acid (HCl). The mixture was incubated at 37°C in a water bath for 10 min. The oil sample was diluted with n-hexane (V/4 V) in order to enhance contact between it and the working FRAP reagent. An aliquot of 300 *μ*L of this diluted oil sample was added to 2 mL of FRAP reagent then made up to volume 10 mL with redistilled water. The reaction mixture was left to incubate for 30 min at 37°C, then centrifuged at 15000 rpm for 10 min to remove solids. A blue-colored compound (Fe (II)-tripyridyltriazine) was formed from the colorless oxidized form Fe (III) by the action of electron-donating antioxidants in the oil sample. The absorbance was measured at 593 nm with a spectrophotometer (Thermo Fisher Scientific Multiskan Go) against a blank reading sample of 2 mL of FRAP reagent made up to 10 mL with redistilled water. A standard curve was prepared using various increasing concentrations (0.005-0.050 mM) of fresh FeSO_4_; 7H_2_O and used on the same day of the preparation. All the measurements were done in triplicate. The FRAP value was expressed as *μ*mol Fe^2+^/kg oil.

### 2.4. Total Phenolic Content

Safflower oil total phenolic content was assessed using the Folin-Ciocalteu method as described [61]. Absorbance was determined at 765 nm against a blank. Measurements were performed in triplicate. Gallic acid was used as a standard. Total phenolics were expressed as gallic acid equivalents per g of oil (GA eq/g oil).

### 2.5. Total Flavonoid Content

Total flavonoid content in safflower seed oil was assayed as described [62] and slightly modified [57]. 1.5 mL of 2% aluminium trichloride solution (AlCl_3_) was added to 1.5 mL DMSO dissolved oil. The mixture was left to incubate in the dark for 30 min at room temperature. The absorbance was determined at 430 nm against a blank. The measurements were performed in triplicate. Quercetin was used as a standard. Total flavonoids in the tested oil were expressed as mg of quercetin equivalents per g of oil (mg Q eq/g oil).

### 2.6. Total Carotenoid and Chlorophyll Contents

Total carotenoid and chlorophyll contents were determined by the colorimetric method as described [63]. Safflower seed oil (1.5 g) was dissolved in 5 mL cyclohexane. The maximum absorption was measured at the wavelength of 470 nm that corresponds to the carotenoid fraction and at 670 nm which corresponds to the chlorophyll fraction. The amounts of the two fractions were calculated using the following formulae:

(2) $$\begin{array}{c} \text{Carotenoid }\left(\text{mg}/\text{kg}\right)=\frac{A_{470}\times 10^{6}}{2.000}\times 100\times \left.\;d\right),\\ \text{Chlorophyll }\left(\text{mg}/\text{kg}\right)=\frac{A_{670}\times 10^{6}}{613}\times 100\times \left.\;d\right), \end{array}$$

where *A* is the absorbance, *d* is the spectrophotometer cell thickness = 1 cm, “2.000” is the specific extinction coefficient of lutein (xanthophyll, major carotenoid fraction component), and “613” is the specific extinction of chlorophyll a (pheophytin, major chlorophyll fraction component).

### 2.7. In Vitro Antimicrobial Assays

#### 2.7.1. Test Microbial Strains

Safflower extracted oil was tested against 10 human pathogenic microorganisms obtained from our collection at the Laboratory of Mycology, Pathologies and Biomarkers (Faculty of Sciences of Tunis, University of Tunis El Manar, Tunisia). This collection was obtained after carrying out isolation and rigorous identification from Tunisian patients' clinical samples: 2 gram-negative bacterial strains (*Escherichia coli* and *Enterobacter cloacae*), 2 gram-positive bacterial strains (*Staphylococcus aureus* and *Streptococcus agalactiae*), 3 yeast species strains (*Candida albicans*, *Candida parapsilosis*, and *Candida sake*), and 3 fungi species (*Aspergillus niger*, *Penicillium digitatum*, and *Fusarium oxysporum*). Antibiotic Ceftazidime (*CAZ30*) and antifungals Voriconazole (VCZ) and Amphotericin B served as reference drugs.

#### 2.7.2. Detection of Antimicrobial Activity

Antibacterial and antifungal assays were performed by the agar well diffusion method [64] and broth microdilution method using sterile Mueller–Hinton media (Bio-Rad, France) for bacterial strains and potato dextrose agar (Bio-Rad, France) for antifungal tests. A fresh cell suspension (0.1 mL) adjusted to 10^7^ CFU/mL for bacteria and 10^6^ spores/mL for fungus was inoculated onto the surface of agar plates. Thereafter, wells with 6 mm in diameter were punched in the inoculated agar medium with sterile Pasteur pipette, and 50 *μ*L of the dissolved oil was added to each well. Negative controls consisted of 50 *μ*L DMSO, used to dissolve the oil (1 V/2 V). The plate was allowed to stand for 2 h to permit the diffusion of the oil followed by incubation at 37°C for 24 h for bacterial strains and 72 h for fungi at 28°C. The antimicrobial activity was evaluated by measuring the inhibition zones (clear areas around the wells) against the test microorganisms. All tests were carried out in triplicate.

#### 2.7.3. Determination of Minimum Inhibitory Concentration (MIC) and Minimal Bactericidal Concentration (MBC)

The minimum inhibitory concentration (MIC) of the tested oil against six bacterial strains was determined accordingly to the microdilution broth method as described [64]. MIC was estimated visually (absence of turbidity) with 3 independent measurements. Minimal bactericidal concentration (MBC) was determined from the microdilution plates used in the MIC assay, according to [65] with modifications [66]. Aliquots (10 *μ*L) of each well without visible growth were transferred to TSA plates, incubated at 36°C for 24 h, and colony growth was verified as previously reported [67].

#### 2.7.4. Lysozyme Activity

The lysozyme activity of the oil was assayed at 1/4 (*v*/*v*) by incubation at 37°C with pathogenic gram-positive bacteria: *Staphylococcus aureus* and *Streptococcus agalactiae*.

The mixed reaction was containing 0.1 mL of diluted oil (1/4) and 0.1 mL of suspension bacterial cell prepared in phosphate buffer after incubation for 60 min at 37°C. The activity was tested turbidimetrically by measuring the decrease in absorbance at 660 nm of the bacterial strain suspensions [66, 67]. Data were the average of three replications and were expressed in arbitrary units per mL (AU/mL).

#### 2.7.5. Determination of Bactericidal Activity

The antimicrobial activity of the tested oil was expressed in arbitrary units per mL (AU/mL), and it was determined by an agar diffusion assay [68]. Briefly, a serial twofold dilution of oil in DMSO and 50 *μ*L of each dilution were spotted onto a TSB agar soft plate seeded with 10^7^ CFU/mL of *Staphylococcus aureus*. The AU/mL was calculated as

(3) $$\begin{array}{c} \text{AU}/\text{mL}=1000\times \frac{D}{A}, \end{array}$$

where *A* is the volume of the tested oil spotted on an agar plate (50 *μ*L in this case) and *D* is the reciprocal of the highest dilution showing a clear inhibition of the indicator strain.

#### 2.7.6. Oil Effects on Viability and Morphology of Fungal Spores

All tested fungi were grown at 28°C on PDA for 10 to 15 days. Sterile water (20 mL) was added to each plate, and the surface was softly scraped with a sterile loop to release the spores. The resulting fungal spore suspension was filtered through a sterile 30 *μ*m filter to remove the mycelial fragments. The conidial suspension of each fungus was adjusted to a concentration of 10^5^ spores/mL by counting with a haemocytometer. To investigate the effects of the described oil on spore germination, 50 *μ*L of conidial suspensions (10^5^ spores/ml) and 50 *μ*L of the oil (1 : 2, *v*/*v*) were pipetted into an Eppendorf tube containing 1 mL with 5% glucose in sterile distilled water; the mix was then incubated at 25°C for 24 h. Control tubes were inoculated only with fungal spores of each tested fungus. Spore's germination inhibition percentage (*I*%) was determined by microscopic examination of spores in the presence of the oil (*E*), compared to the control tube containing only the spore suspensions (*C*) [69]. The used formula was

(4) $$\begin{array}{c} I\;\left(\%\right)\text{: }\frac{C-E}{C}\times 100. \end{array}$$

To determine the effect of the tested oil on the morphological spore's germination, 100 *μ*L from each tube was directly observed with optical microscopy (CETI) at 40x.

### 2.8. Statistical Analysis

The statistical data analysis was performed using SPSS statistics 20.0 (SPSS Inc., Chicago, Illinois, USA) followed by *t*-test to assess the effect of treatments. The results were expressed as mean values ± SEM. A difference was considered significant if *p* < 0.05.

## 3. Results

### 3.1. Physicochemical Properties of Safflower Seed Oil

The physicochemical properties of the tested oil are reported in Table 1. The moisture ratio of the dried seeds was of 4.71 ± 0.03%. Safflower seed oil extracted by first cold pressing is a bright yellowish-amber fluid oil with characteristic vegetal smell. It is a natural, free of solvents, dry, and noncomedogenic oil. It has a density of 0.921 ± 0.002 at 20°C (g/cm3), a refractive index at 20°C of 1.477 ± 0.001, a saponification value of 191.2 ± 0.350 mg KOH/g oil, an iodine index of 137.5 ± 0.450 g *I*_2_/100 g oil, a peroxide value of 1.985 ± 0.043 meq O_2_/kg oil, and an acidity index of 1.523 ± 0.041 mg KOH/g oil.

### 3.2. Antioxidant Activities of Safflower Seed Oil

The results have shown that safflower seed oil exhibited high scavenging effects against DPPH and ABTS free radicals. We registered a percentage of inhibition compared to Vit. C 89.41 ± 0.38 Vit. C eq/g oil and 88.52 ± 0.45 Vit. C eq/g oil, respectively. The FRAP value of safflower seed oil was estimated at 247.5 ± 0.034 *μ*mol Fe^2+^/kg oil (Table 2).

### 3.3. Total Phenolic, Total Flavonoid, Total Carotenoid, and Total Chlorophyll Contents

Table 3 presents the data of the total phenolic, total flavonoid, total carotenoid, and total chlorophyll amounts measured in the safflower oil sample. They were, respectively, of 98.52 ± 0.80 GA eq/g oil, 35.79 ± 0.34 Q eq/g oil, 18.43 ± 0.020 mg/kg oil, and 3.9 ± 0.10 mg/kg oil.

### 3.4. Antimicrobial Activity of Safflower Seed Extracted Oil

#### 3.4.1. Antibacterial Activity

The tested oil dissolved in DMSO at 1/3 (*v*/*v*) proved high antibacterial activity since it was active against three bacterial species *Escherichia coli*, *Streptococcus agalactiae*, and *Enterobacter cloacae* from a total of four bacterial species. The diameter inhibition values vary with the bacterial species from 13.0 mm to 15.0 mm (Table 4).

In order to determine the bacteriostatic and/or bactericidal effects of the described oil, a volume from each well without visible growth was transferred to TSA plates, incubated at 37°C for 24 h, and colony growth was examined. The obtained results showed that our oil has a bacteriostatic effect against all tested bacterial species since we observed a bacterial colony after incubation. MIC and MBC obtained values were, respectively, 1/16 and 1/32 for *Escherichia coli* and *Streptococcus agalactiae*.

**Figure 1:**
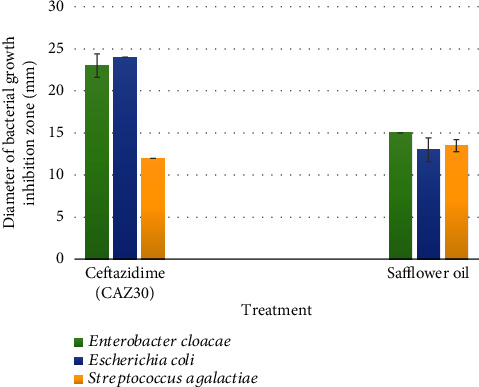
The antibacterial effect of safflower oil on used bacterial species compared to the antibiotic used standard Ceftazidime (CAZ30). Values measured represent the diameters of bacterial growth inhibition zone expressed in mm. Safflower oil showed lower bactericidal activity against *Escherichia coli* and *Enterobacter cloacae* than Ceftazidime (CAZ30). However, its effect against *Streptococcus agalactiae* was greater than that of CAZ30.

The highest antibacterial activity was observed against *Enterobacter cloacae* by giving the maximum diameter of inhibition of 15 mm and the high MIC and MIB values, respectively, of 1/32 and 1/64. This data was also confirmed by its most activity than the antibiotic used here Ceftazidime (CAZ30) compared to other antibacterial activity against other species which were less than those exhibited by Ceftazidime (CAZ30) (Figure 1).

The bactericidal activity expressed in AU/mL was of 320 AUmL^−1^ against *Escherichia coli* and *Streptococcus agalactiae* and 640 against *Enterobacter cloacae* (Table 4).

**Figure 2:**
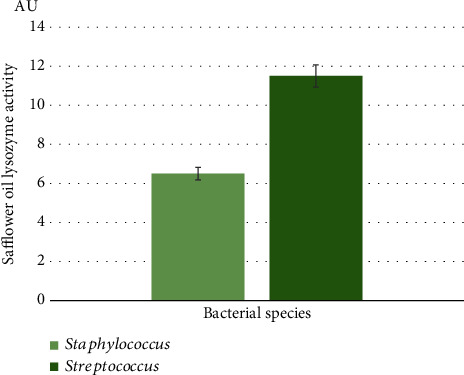
Lysozyme activity of safflower oil tested at 1/4 (*v*/*v*) by incubation at 37°C with pathogenic gram-positive bacteria: *Streptococcus agalactiae* and *Staphylococcus aureus*. Data are the average of three replications, and bars present the standard error of the means. A lysozyme activity was greater on *Streptococcus agalactiae* rather than on *Staphylococcus aureus*.

A low value of lysozyme activity was also detected against two tested gram-positive pathogenic strains *Streptococcus agalactiae* and *Staphylococcus aureus*. We found 11.5 and 6.5 AU, respectively (Figure 2).

#### 3.4.2. Antifungal Activity

The obtained results have shown that safflower oil exhibited a significant antifungal activity against two *Candida* species, with a diameter zone inhibition of 15.5 mm for *Candida parapsilosis* and 15.0 mm for *Candida sake*. No growth-inhibitory action on *Candida albicans* was observed. Fungicidal activity was obtained on the three fungal species (*Aspergillus niger*, *Penicillium digitatum*, and *Fusarium oxysporum*) with an inhibition zone diameter of ranging from 11 to 12.5 mm (Table 5).

**Figure 3:**
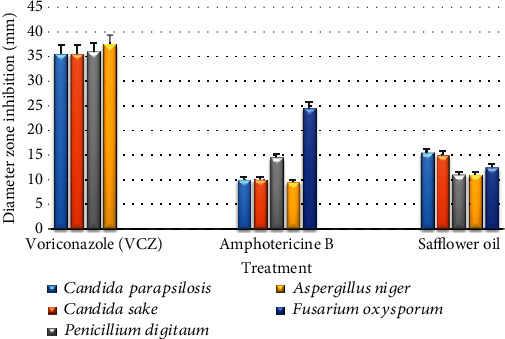
Comparison of the antifungal activities between antifungal drugs and safflower oil on the tested fungal species. Values measured represent diameters of fungal growth inhibition zone (mm). Voriconazole (VCZ) and Amphotericin B were used as positive controls. Safflower oil treatment significantly reduced the percentage of spores' germination and branching of the germ tubes of the tested fungal strains.

Comparing the antifungal activity of safflower oil with that of two antifungal drugs, we could note that it has antifungal effects on all the tested fungal strains. Its effectiveness was quite similar to that of Amphotericin B against *Aspergillus niger*. Nonetheless, its antifungal efficiency was weaker than that of Voriconazole (VCZ) except for *Fusarium oxysporum* where safflower exhibited antifungal effect as well as Amphotericin B where VCZ had no effect on this strain (Figure 3).

From our data, it appeared that safflower oil was able to reduce spores' germination of the three tested fungi with a high percentage of inhibition of 84.8% for *Aspergillus niger* and more than 88% for the other tested fungi, compared to untreated controls.

**Figure 4:**
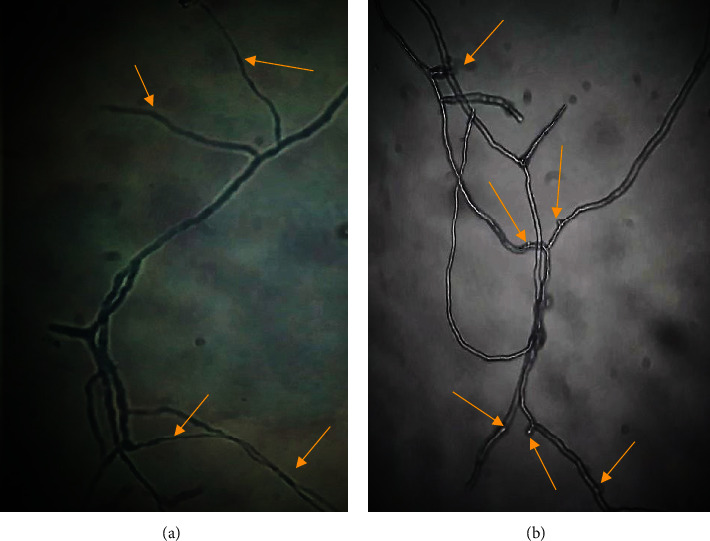
Microscopic morphology (×400) of A*spergillus niger* spores' germination under normal conditions (a) and after treatment with safflower oil (b). The arrows indicate the spores' germinative tubes. Safflower oil reduced markedly the length of *Aspergillus niger* spores' germination tube compared to the controls.

Microscopic observation of spore germination morphology of conidial suspensions treated with safflower oil at 1/4 (*v*/*v*) compared to conidial suspension in the absence of the oil has shown a marked reduction of the length of the germinative tube and the ramification rate of the spores. For instance, Figure 4 shows the observation of *Aspergillus niger*'s spore germination in the absence of any treatment (Figure 4(a)) and after treatment with safflower oil (Figure 4(b)). Similar effects were observed for *Penicillium digitatium* and *Fusarium oxysporum* spores.

## 4. Discussion

Our results have shown that the oil extracted from safflower seeds under cold pressing exhibited high antioxidant activities through its scavenging activities on free radicals DPPH and ABTS and its strong ferric reducing antioxidant power (FRAP). Moreover, we registered notable antimicrobial effects of safflower oil against human skin opportunistic pathogenic bacteria, yeast, and fungi, which commonly alter the healing of skin wounds. These properties would allow the tested oil to promote the healing process of cutaneous injuries by topical application.

Skin injury can be considered as an oxidative stress-inducing factor. The wound repair process requires an increase in the consumption of oxygen O_2_ which is a paramount factor in the mitochondrial production of adenosine triphosphate (ATP), the energetic molecule used by all actors in the healing process, especially during the early phases, hemostasis and inflammation. Although the rupture of the blood vessels caused by the skin injury induces a state of hypoxia, it seems that the lack of O_2_ starts to jump the healing process via the release of HIF-1 [70] and huge amounts of oxygen reactive species (ROS) in the wound site [34, 71]. Oxygen- (O_2_-) derived molecules are known as reactive oxygen species (ROS) such as H_2_O_2_.

When the platelets and neutrophils are exposed to the cutaneous ECM and collagen after the capillary rupture, they get activated and release chemical mediators in the wound bed and a large amount of ROS. At homeostatic low doses, these compounds are beneficial and intervene since the first minutes after skin trauma in several ROS-signaling pathways [35, 36]. First of all, ROS stimulate the constriction of the vessels surrounding the wound to reduce blood loss, in parallel with the activation of the hemostatic cascade, the formation of thrombin, and as a result a thrombus that can stop local hemorrhage.

They also provide further signals within the intercellular network that supports the wound healing process and activate the recruitment of immunocytes to migrate to the wound site to fight invading pathogens [72, 73]. It has been reported that during the inflammatory phase, neutrophils intervene first, because of their abundance in the blood, followed by some innate immune cells like mast cells and dendritic epidermal T cells (DETCs) and by monocytes which then differentiate into mature macrophages which are strongly activated in the wound bed during the acute inflammatory process. These cells then release a plethora of interacting molecules, active mediators such as chemotactic, and growth factors, cytokines, and chemokines (IL-1, IL-6, VEGF, TNF-*α*,…) with proinflammatory and proteolytic enzymatic properties [71–73]. PDGF and TGF-*β* are released by the recruited platelets to attract more platelets, fibroblasts, and inflammatory cells. TNF-*α* has been reported to be an essential factor in promoting centripetal migration of keratinocytes from the edges to the center of the wound site and allowing reepithelialization and closure of the epidermis [74].

Furthermore, other amounts of ROS are released during phagocytosis to stunt bacterial and fungus proliferation via bacteriostatic and fungistatic actions. Skin wounds get sometimes worse if not well managed, especially under bad care conditions that lead to infections and the extension of the inflammatory phase, with an enhancement of ROS and free radical production which are detrimental to wound healing [36, 70]. In addition, it should be noted that under normal healing conditions, endogenous antioxidants such as SOD, GPx, and Trx-1 and Trx-2 are secreted and released in the wound bed to counteract the oxidative stress damages induced by the free radicals produced during the inflammatory phase [36]. It is therefore the physiologic balance between prooxidant factors and antioxidant agents that would be one of the keys to efficient tissue repair. But in case of reduction of secretion of endogenous antioxidants in certain patients especially the elderly with underlying pathologies such as immunological depressed status, diabetes mellitus, hemiplegia, and tetraplegia that impair autonomic mobility, the damages caused by oxidative stress are amplified, leading to wound infections, delayed healing, and chronicity of wounds. This is of a great concern in nursing and wound care.

Supplementation by topical application on the wound bed with exogenous compounds which have antioxidant potential, such as safflower oil, would be an effective strategy in wound management. The antioxidant properties of safflower oil could be attributed to its richness in several bioactive compounds such as polyphenols, flavonoids, carotenoids, and chlorophylls which were detected in our oil extract. In fact, various studies have proven the very potent antioxidant potential of these compounds [69, 71–73] and their protective effects in human health against oxidative-stress-induced diseases and physiological disruptions [75–77]. Moreover, it has been shown that safflower seeds' oil is particularly rich in *α* tocopherol-vitamin E [78] and in phytosterols such as campesterol, stigmasterol, and *β*-sitosterol which have strong potential antioxidant effects [79]. Topical management of skin wounds by this oil could promote the healing process as it is mainly composed at a high percentage by unsaturated fatty acids [80] especially linoleic acid (C18:2n-6) and oleic acid (C18:1 n-9) which are important components of the cell membranes [81].

Additionally, it is known that linoleic acid is a precursor in the synthesis pathways of several bioactive mediators (thromboxanes, prostaglandins, and leukotrienes) which are very active in the neoangiogenesis and dermal regeneration [82]. In addition, the tested oil was described to be very rich in phospholipids such as phosphatidylethanolamine, phosphatidylcholine, and phosphatidylinositol which are major components of the cell membranes. So, safflower oil may provide crucial lipid elements for the genesis of the cells which are newly formed during the healing process [83, 84, 85].

Another criterion seems to be necessary during the early stages of the healing process, namely, a good level of hydration in the wound. In some cases like deep wounds, burns, bedsores, and skin ulcers, wound management requires the use of a variety of products such as hydrogel dressings that provide both hydration and the removal of debris and waste from the wound bed. Safflower extracted oil could ensure a good level of wound hydration by providing an insulating barrier between the external environment and the exudate in the wound bed, thus preserving it from dryness and promoting the effectiveness of the healing actors.

Our study showed that the oil extracted by cold pressing from safflower seeds had high antibacterial activities by both bacteriostatic and bactericidal ways of action against the tested pathogenic bacterial strains (*Enterobacter cloacae*, *Escherichia coli*, and *Streptococcus agalactiae*). Under the same conditions of extraction and experimentation, *Opuntia ficus indica* oil was less efficient than safflower oil as we noted antibacterial activity only against *Enterobacter cloacae* [66]. An antifungal potential against the fungal-tested strains (*Candida parapsilosis*, *Candida sake*, *Aspergillus niger*, *Penicillium digitatum*, and *Fusarium oxysporum*) was noted. However, no activity has been detected against *Candida albicans* and *Staphylococcus aureus* based on diameter inhibition zone measurement. On another hand, we registered a lysozyme activity. Due to its muramidase activity, lysozyme has long been known to exert its antimicrobial action by specially hydrolyzing the 1,4-D-linkage between N-acetylmuramic acid and N-acetyl-D-glucosamine of cell wall peptidoglycan which is the major component of the gram-positive bacterial cell wall [86], hence inducing bacterial lysis and providing some protection against bacterial infection. It is likely oil lysozyme activity against the tested *Staphylococcus aureus* strain was not sufficient to inhibit its growth on the agar plates. This lysozyme activity of safflower oil may enhance its potency to inactivate some bacterial strains and allows the understanding of one of its ways of action.

Our results corroborate other findings on the antimicrobial effects of *Carthamus tinctorius*. A recent study showed that methanolic and aqueous extracts from the seeds of this species had antibacterial activities against *Escherichia coli* and *Acinetobacter baumanii* with inhibition diameters of 3 mm and 5 mm, respectively, while based on MICs, the same extracts exhibited antibacterial effects against *Escherichia coli*, *Staphylococcus aureus*, *Acinetobacter baumanii*, *Klebsiella pneumonia*, and *Pseudomonas aeruginosa* [87]. In addition, it has been proven that flower extracts from *Carthamus tinctorius L.* harvested at the latter stage of flowering have significant antimicrobial effects against a fungal strain (*Candida albicans*) and some bacterial strains (*Escherichia coli* (ATCC 25218), methicillin-resistant *Staphylococcus aureus* (ATCC 25923), *Pseudomonas aeruginosa* (ATCC 27853), *Bacillus cereus* (ATCC 14759)). The most potent activity has been against *Escherichia coli* with an inhibition zone of 26 mm [87].

Several studies outlined that antimicrobial activities of plant extracts are due to their phenolic contents which cause cell membrane disruption, thus inducing cytoplasmic element spillage and cell necrosis [88–90]. Safflower oil phenolics may contribute by this way to inactivate bacterial growth. Moreover, as it is rich with polyunsaturated fatty acids (oleic and linoleic acids), safflower oil could act against both bacteria and fungi. Fatty acids were reported to inhibit some membrane enzymes like glucosyltransferase and to activate autolytic cell wall enzymes, leading to cell death (bactericidal or fungicidal effects). Moreover, they were cited to reduce energy pathway production in the mitochondria, hence inhibiting bacterial (bacteriostatic effect) or fungal growth (fungistatic action) [87]. This is in line with our results since we found a slowdown in fungal spore germination. In addition, these fatty acid-related antimicrobial effects could be combined with those of the phytosterols present in safflower seed extracted oil to enhance its effectiveness against microbial infections.

## 5. Conclusion

The present study revealed that the oil extracted by cold pressing from the seeds of safflower (*Carthamus tinctorius L.*) exhibited high antioxidant effects and antimicrobial potentialities against several opportunistic skin pathogens. It seemed to act by bacteriostatic and bactericidal pathways as well as a strong antifungal growth inhibition.

A judicious strategy for the management of acute and especially chronic skin injuries would be to administer topically a multitherapy composed of antioxidants, one or more antimicrobials (antibiotics and antifungals depending on the patient's condition), and skin regenerating and restructuring compounds, safe from health side effects. The bioactive components of *Carthamus tinctorius L.* seed oil extracted by cold pressing may allow it to be considered as a good alternative natural therapeutic for the management of skin injuries, and the prevention of skin infections.

## Figures and Tables

**Table 1:** Physicochemical properties of first cold pressed safflower seed oil.

Parameters	
Physical state at room temperature	Liquid
Colour	Bright yellowish-amber
Odour	Characteristic vegetal smell
Texture	Dry oil
Property	Fluid, noncomedogenic
Density at 20°C	0.921 ± 0.002
Refractive index at 20°C	1.477 ± 0.001
Saponification value (mg KOH/g oil)	191.2 ± 0.350
Iodine index (g *I*_2_/100 g oil)	137.5 ± 0.450
Peroxide value (meq O_2_/kg oil)	1.985 ± 0.043
Acidity index (mg KOH/g oil)	1.523 ± 0.041

^∗^Values given are the means of three measurements ± standard error.

**Table 2:** Antioxidant activities of safflower seed oil.

Scavenging activity against DPPH radical % inhibition (Vit. C eq/g oil)	Scavenging activity against ABTS radical % inhibition (Vit. C eq/g oil)	FRAP value (*μ*mol Fe^2+^/kg oil)
89.41 ± 0.38	88.52 ± 0.45	247.5 ± 0.034

^∗^Values given are the means of three measurements ± standard error.

**Table 3:** Total phenolic, total flavonoid, total carotenoid, and total chlorophyll contents of safflower seed oil.

Total phenolics (GA eq/g oil)	Flavonoids (mg Q eq /g oil)	Carotenoids (mg/kg oil)	Total chlorophylls (mg/kg oil)
98.52 ± 0.80	35.79 ± 0.34	18.43 ± 0.020	3.9 ± 0.010

^∗^Values given are the means of three measurements ± standard error.

**Table 4:** Antibacterial activity of safflower seed oil on the tested bacterial strains.

Microorganisms	MIC	MBC	Diameter zone inhibition (mm)	Bactericidal activity (AU/mL)
Bacteria				
*Escherichia coli*	1/16	1/32	13.0 ± 1.4	320
*Streptococcus agalactiae*	1/16	1/32	13.5 ± 0.7	320
*Enterobacter cloacae*	1/32	1/64	15.0 ± 0.0	640
*Staphylococcus aureus*	-	-	-	-

Values given are the means of three measurements ± standard error. -: absence of activity.

**Table 5:** Antifungal activity of safflower seed oil on the tested fungal species.

Microorganisms	Diameter zone inhibition (mm)
Yeasts	
*Candida albicans*	-
*Candida parapsilosis*	15.5 ± 0.7
*Candida sake*	15.0 ± 1.4
Fungi	
*Aspergillus niger*	11.0 ± 1.4
*Penicillium digitatum*	11.0 ± 0.0
*Fusarium oxysporum*	12.5 ± 0.7

Values given are the means of three measurements ± standard error. -: absence of activity.

## Data Availability

The data used to support the findings of this study are available from the corresponding author upon request.

## Abbreviations

CFU: Colony-forming unit
DMSO: Dimethylsulfoxide
MIC: Minimum inhibitory concentration
MBC: Minimal bactericidal concentration
PDA: Potato dextrose agar
TSA: Trypticase soy agar
TSB: Trypticase soy broth
AU/mL: Arbitrary unit per mL
FRAP: Ferric reducing antioxidant power
PDGF: Platelet-derived growth factor
TNF-*α*: Tumor necrosis factor alpha
IL-1: Interleukin 1
IL-6: Interleukin 6
VEGF: Vascular endothelial growth factor
EGF: Epidermal growth factor
FGF: Fibroblast growth factor
TGF: Transforming growth factor
KGF: Keratinocyte growth factor
SOD: Superoxide dismutase
GPx: Glutathione peroxidase
Trx-1: Thioredoxin-1
Trx-2: Thioredoxin-2.

## References

[1] Wang P.-H., Huang B.-S., Horng H.-C., Yeh C. C., Chen Y. J. (2018). Wound healing. *Journal of the Chinese Medical Association*.

[2] Lopez F. A., Lartchenko S. (2006). Skin and soft tissue infections. *Infectious Disease Clinics of North America*.

[3] Lobo V., Patil A., Phatak A., Chandra N. (2010). Free radicals, antioxidants and functional foods: impact on human health. *Pharmacognosy Reviews*.

[4] Asadi M., Alamdari D. H., Rahimi H. R. (2014). Treatment of life-threatening wounds with a combination of allogenic platelet-rich plasma, fibrin glue and collagen matrix, and a literature review. *Experimental and Therapeutic Medicine*.

[5] Oshikata C., Tsurikisawa N., Saito A. (2013). Fatal pneumonia caused by Penicillium digitatum; a case report. *BMC Pulmonary Medicine*.

[6] Ovington L. (2003). Bacterial toxins and wound healing. *Ostomy/ wound management*.

[7] Edwards R., Harding K. G. (2004). Bacteria and wound healing. *Current Opinion in Infectious Diseases*.

[8] Da Silva L. P., Reis R. L., Correlo V. M., Marques A. P. (2019). Hydrogel-based strategies to advance therapies for chronic skin wounds. *Annual Review of Biomedical engineering*.

[9] Allman R.-M., Goode P.-S., Burst N.-B.-S., Bartolucci A.-A., Thomas D.-R. (1999). Pressure ulcers, hospital complications and disease severity: impact on hospital costs and length of stay. *Advances in skin & wound care*.

[10] Gist S., Tio-Matos I., Falzgraf S., Cameron S., Beebe M. (2009). Wound care in the geriatric client. *Clinical Interventions in Aging*.

[11] Webster J., Scuffham P., Stankiewicz M., Chaboyer W.-P. (2014). Negative pressure wound therapy for skin grafts and surgical wounds healing by primary intention. *Cochrane Database of Systematic Reviews*.

[12] Pino A.-E., Taghya S., Chapman C., Bowker J.-H. (2011). Lower-limb amputations in patients with diabetes mellitus. *Orthopedics*.

[13] Ruppen C., Notter J., Strahm C., Sonderegger B., Sendi P. (2018). Osteoarticular and skin and soft-tissue infections caused by Streptococcus agalactiae in elderly patients are frequently associated with bacteremia. *Diagnostic Microbiology and Infectious Disease*.

[14] Muto C. A., Jernigan J. A., Ostrowsky B. E. (2003). SHEA guideline for preventing nosocomial transmission of multidrug-resistant strains of Staphylococcus aureus and Enterococcus. *Infectious Control & Hospital epidemiology*.

[15] Chiller K., Selkin B. A., Murakawa G. J. (2001). Skin microflora and bacterial infections of the skin. *Journal of Investigative dermatology Symposium Proceedings*.

[16] Babic M. N., Zalar P., Zenko B., Schroers H. J., Dzeroski S., Gunde-Cimerman N. (2015). Candida and Fusarium species known as opportunistic human pathogens from customer-accessible parts of residential washing machines. *Fungal Biology*.

[17] Mangoni M. L., Mc Dermott A. M., Zasloff M. (2016). Antimicrobial peptides and wound healing: biological and therapeutic considerations. *Experimental Dermatology*.

[18] Amsler K. M., Davies T. A., Shang W., Jacobs M. R., Bush K. (2008). In vitro activity of ceftobiprole against pathogens from two phase 3 clinical trials of complicated skin and skin structure infections. *Antimicrobial Agents and Chemotherapy*.

[19] Van der Mee-Marquet N., Fourny L., Arnault L. (2008). Molecular characterization of human-colonizing Streptococcus agalactiae strains isolated from throat, skin, anal margin, and genital body sites. *Journal of Clinical Microbiology*.

[20] Ekpo M. A., Etim P. C. (2009). Antimicrobial activity of ethanolic and aqueous extracts of Sida acuta on microorganisms from skin infections. *Journal of Medicinal Plants Research*.

[21] Petkovsek Z., Elersic K., Gubina M., Zgur-Bertok D., Erjavec M. S. (2010). Virulence potential of Escherichia coli isolates from skin and soft tissue infections. *Journal of Clinical Microbiology*.

[22] Zanardi I., Burgassi S., Paccagnini E., Gentile M., Bocci V., Travagli V. (2013). What is the best strategy for enhancing the effects of topically applied ozonated oils in cutaneous infections?. *BioMed Research International*.

[23] James G. A., Swogger E., Wolcott R. (2008). Biofilms in chronic wounds. *Wound Repair and Regeneration*.

[24] Omar A., Wright J. B., Schultz G., Burrel R., Nadworny P. (2017). Microbial biofilms and chronic wounds. *Microorganisms*.

[25] Malone M., Bjarnsholt T., McBain A. J. (2017). The prevalence of biofilms in chronic wounds: a systematic review and meta-analysis of published data. *Journal of Wound Care*.

[26] Weller R., Price R. J., Ormerod A. D., Benjamin N., Leifert C. (2001). Antimicrobial effect of acidified nitrite on dermatophyte fungi, Candida and bacterial skin pathogens. *Journal of Applied Microbiology*.

[27] Angaman R. K., Orsot B. M. A. B., Camara D., Abo K., Zirihi N. G. (2018). Etude ethnobotanique de plantes de la flore du Département d’Abengourou, en Côte d’Ivoire et évaluation in vitro de l’activité antifongique d’extraits de Terminalia superba Engl. Diels sur deux espèces de champignons, Aspergillus niger Van Tieghem et Fusa. *International Journal of Biological and Chemical Sciences*.

[28] Brink A. J., Richards G. A. (2020). The role of multidrug and extensive-drug resistant Gam-negative bacteria in skin and soft tissue infections. *Current Opinion in Infectious Diseases*.

[29] Muller A., Patry I., Talon D. (2006). Mise en place d'un outil informatisé de surveillance de la résistance bactérienne et de la consommation antibiotique dans un centre hospitalier universitaire. *Pathologie-Biologie*.

[30] Klevens R. M., Morrison M. A., Nadle J. (2007). Invasive methicillin-resistant Staphylococcus aureus infections in the United States. *Journal of the American Medical Association*.

[31] White R. J., Cutting K., Kingsley A. (2006). Topical antimicrobials in the control of wound bioburden. *Ostomy/Wound Management*.

[32] Guérin F. (2015). Infections à Enterobacter cloacae complex: résistance aux antibiotiques et traitement. *Journal des Anti-infectieux*.

[33] Carbone C., Teixeira M. D. C., Sousa M. D. C. (2019). Clotrimazole-loaded Mediterranean essential oils NLC: a synergic treatment of Candida skin infections. *Pharmaceutics*.

[34] Werner S., Grose R. (2003). Regulation of wound healing by growth factors and cytokines. *Physiological Reviews*.

[35] Kanta J. (2011). The role of hydrogen peroxide and other reactive oxygen species in wound healing. *Acta Medica (Hradec Kralove)*.

[36] Dunnill C., Patton T., Brennan J. (2017). Reactive oxygen species (ROS) and wound healing: the functional role of ROS and emerging ROS-modulating technologies for augmentation of the healing process. *International Wound Journal*.

[37] Wikaningtyas P., Sukandar E. Y. (2016). The antibacterial activity of selected plants towards resistant bacteria isolated from clinical specimens. *Asian Pacific Journal of Tropical Biomedicine*.

[38] Pawar R. S., Kumar S., Toppo F. A., Lakshmi P. K., Suryavanshi P. (2016). Sida cordifolia Linn. accelerates wound healing process in type 2 diabetic rats. *Journal of Acute Medicine*.

[39] Bijauliya R. K., Alok S., Kumar M., Chanchal D. K., Yadav S. (2017). A comprehensive review on herbal cosmetics. *International Journal of Pharmaceutical Sciences and Research*.

[40] Henriques A., Jackson S., Cooper R., Burton N. (2006). Free radical production and quenching in honeys with wound healing potential. *Journal of Antimicrobial Chemotherapy*.

[41] Panchatcharam M., Miriyala S., Gayathri V. S., Suguna L. (2006). Curcumin improves wound healing by modulating collagen and decreasing reactive oxygen species. *Molecular and Cellular Biochemistry*.

[42] Farahpour N., Mirzakhani J. (2015). Hydroethanolic Pistacia atlantica hulls extract improved wound healing process; evidence for mast cells infiltration, angiogenesis and RNA stability. *International Journal of Suregey*.

[43] Thangapazham R. L., Sharad S., Maheshwari R. K. (2016). Phytochemicals in wound healing. *Advances in Wound Care*.

[44] Karimzadeh S., Farahpour M. R. (2017). Topical application of Salvia officinalis hydroethanolic leaf extract improves wound healing process. *Indian Journal of Experimental Biology*.

[45] Manzuoerh R., Farahpour M. R., Oryan A., Sonboli A. (2019). Effectiveness of topical administration of Anethum graveolens essential oil on MRSA-infected wounds. *Biomedicine & Pharmacotherapy*.

[46] Li C., Yao Y., Zhao G. (2011). Comparison and analysis of fatty acids, sterols, and tocopherols in eight vegetable oils. *Journal of Agricultural and Food Chemistry*.

[47] Djerrou J., Maameri Z., Hamdo-Pacha Y. (2010). Effect of virgin fatty oil of Pistacia lentiscus on experimental burn wound’s healing in rabbits. *African Journal of Traditional,Complementary and Alternative Medicines*.

[48] Farahpour M. R., Fathollahpour S. (2015). Topical co-administration of flaxseed and pistachio ointment promoted wound healing; evidence for histopathological features. *Comparative Clinical Pathology*.

[49] Khedir S. B., Bardaa S., Chabchoub N., Moalla D., Sahnoun Z., Rebai T. (2017). The healing effect of Pistacia lentiscus fruit oil on laser burn. *Pharmaceutical Biology*.

[50] Bardaa S., Chabchoub N., Jridi M. (2016). The effect of natural extracts on laser burn wound healing. *Journal of Surgical Research*.

[51] Bardaa S., Halima N. B., Aloui F. (2016). Oil from pumpkin (Cucurbita pepo L.) seeds: evaluation of its functional properties on wound healing in rats. *Lipids in Health and Disease*.

[52] Khémiri I., Essghaier Hédi B., Sadfi Zouaoui N., Ben Gdara N., Bitri L. (2019). The antimicrobial and wound healing potential ofOpuntia ficus indica L. inermisExtracted oil from Tunisia. *Evidence-Based Complementary and Alternative Medicine*.

[53] Laursen R., Mouri C. (2013). Decomposition and analysis of carthamin in safflower-dyed textiles. *E-Preservation Science*.

[54] Zhang H. L., Nagatsu A., Watanabe T., Okuyama H. (1997). Antioxidative compounds isolated from safflower (Cartthamus tinctorius L.) oil cake. *Chemical and Pharmaceutical Bulletin*.

[55] Lee J. Y., Chang E. J., Kim H. J., Park J. H., Choi S. W. (2002). Antioxidative flavonoids from leaves ofCarthamus tinctorius. *Archives of Pharmacal Research*.

[56] Salem N., Msaada K., Hamdaoui G., Limam F., Marzouk B. (2011). Variation in phenolic composition and antioxidant activity during flower development of safflower (Carthamus tinctorius L.). *Journal of Agricultural and Food Chemistry*.

[57] Khémiri I., Bitri L. (2019). Effectiveness ofOpuntia ficus indicaL.inermisSeed oil in the protection and the healing of experimentally induced gastric mucosa ulcer. *Oxidative Medicine and cellular Longevity*.

[58] Bendaoud H., Romdhane M., Souchard J. P., Cazaux S., Bouajila J. (2010). Chemical composition and anticancer and antioxidant activities of Schinus molle L. and Schinus terebenthifolius Raddi berries essential oils. *Journal of Food Science*.

[59] Benzie I. F., Strain J. J. (1999). [2] Ferric reducing/antioxidant power assay: direct measure of total antioxidant activity of biological fluids and modified version for simultaneous measurement of total antioxidant power and ascorbic acid concentration. *Methods in enzymology*.

[60] Szydlowska-Czerniak A., Dianoczki C., Recseg K., Karlovits G., Szlyka E. (2008). Determination of antioxidant capacities of vegetable oils by ferric-ion spectrophotometric methods. *Talanta*.

[61] El Kar C., Ferchichi A., Attia F., Bouajila J. (2011). Pomegranate (Punica granatum) juices: chemical composition, micronutrient cations and antioxidant capacity. *Journal of Food Science*.

[62] Bahorun T., Gressier B., Trotin F. (1996). Oxygen species scavenging activity of phenolic extracts from hawthorn fresh plant organs and pharmaceutical preparations. *Arzneimittel-forschung*.

[63] Minguez-Mosquera M. I., Gandul-Rojas B., Montano-Asqueino A., Garrido-Fernandez J. (1991). Dertermination of chlorophylls and carotenoids by high-performance liquid chromatography during olive lactic fermentation. *Journal of Chromatography A*.

[64] Tagg J. R., McGiven A. R. (1971). Assay system for bacteriocins. *Applied Microbiology*.

[65] Celiktas O. Y., Hameskocabas E. E., Bedir E., VardarSukan F., Ozek T., Baser K. H. C. (2007). Antimicrobial activities of methanol extracts and essential oils of Rosmarinus officinalis, depending on location and seasonal variations. *Food Chemistry*.

[66] Khémiri I., Essghaier-Hédi B., Sadfi-Zouaoui N., Ben Gdara N., Bitri L. (2019). The antimicrobial and wound healing potential of Opuntia ficus indica L. inermis extracted oil from Tunisia. *Evidence-Based Complementary and Alternative Medicine*.

[67] Sehimi H., Essghaier B., Barea E., Sadfi-Zouaoui N., Zid M. F. (2019). Synthesis, structural study, magnetic susceptibility and antimicrobial activity of the first (*μ*-oxo)-bis(oxalato)-vanadium(IV) 1D coordination polymer. *Journal of Molecular Structure*.

[68] Graciela M., Vignolo M., De Kairuz N., Aida A. P., De Ruiz H., Oliver G. (1995). Influence of growth conditions on the production of lactocin 705, a bacteriocin produced by Lactobacillus casei CRL 705. *Journal of Applied Bacteriology*.

[69] Sarangi N., Athukorala P., Dilantha Fernando W. G., Rashid K. Y., Kievit T. D. (2010). The role of volatile and non-volatile antibiotics produced by Pseudomonas chlororaphis strain PA23 in its root colonization and control of Sclerotinia sclerotiorum. *Biocontrol Science and Technology*.

[70] Rodriguez P. G., Felix F. N., Woodley D. T., Shim E. K. (2008). The role of oxygen in wound healing: a review of the literature. *Dermatologic Surgery*.

[71] Gurtner G. C., Werner S., Barrandon Y., Longaker M. T. (2008). Wound repair and regeneration. *Nature*.

[72] Zaja-Milatovic S., Richmond A. (2008). CXC chemokines and their receptors: a case for a significant biological role in cutaneous wound healing. *Histology and histopathology*.

[73] Wilgus T. A. (2008). Immune cells in the healing skin wound; influential players at each stage of repair. *Pharmacological Research*.

[74] Li Y., Fan J., Chen M., Li W., Woodley D. T. (2006). Transforming growth factor-alpha: a major human serum factor that promotes human keratinocyte migration. *The Journal of Investigative Dermatology*.

[75] Rice-Evans C., Miller N., Paganga G. (1997). Antioxidant properties of phenolic compounds. *Trends in Plant Science*.

[76] Zielinski H., Kozlowska H. (2000). Antioxidant activity and total phenolics in selected cereal grains and their different morphological fractions. *Journal of Agricultural and Food Chemistry*.

[77] Huan-Xia Z., Hai-Sheng Z., Shu-Fang Y. (2014). Phenolic compounds and its antioxidant activities in ethanolic extracts from seven cultivars of Chinese jujube. *Food Science and Human Wellness*.

[78] Rice-Evans C., Miller N. J., Paganga G. (1996). Structure-antioxidant activity relationships of flavonoids and phenolic acids. *Free Radical Biology and Medicine*.

[79] Erol-Dayi Ö., Arda N., Erdem G. (2012). Protective effects of olive oil phenolics and gallic acid on hydrogen peroxide-induced apoptosis. *European Journal of Nutrition*.

[80] Katkade M. B., Syed H. M., Andhale R. R., Sontakke M. D. (2018). Fatty acid profile and quality assessment of safflower (Carthamus tinctorius) oil. *Journal of Pharmacognosy and Phytochemistry*.

[81] Xican L., Xiaoting W., Ling H. (2009). Correlation between antioxidant activities and phenolic contents of Radix Angelicae Sinensis (Dangugui). *Molecules*.

[82] Samanci B., Ozkaynak E. (2003). Effect of planting date on seed yield, oil content and fatty acid composition of safflower (Carthamus tinctorius) cultivars grown in the Mediterranean region of Turkey. *Journal of Agronomy and Crop Science*.

[83] Nauman K., Rao Sanaullah K., Hussain M. I., Muhammad F., Asif A., Iftikhar A. (2017). A comprehensive characterization of safflower oil for its potential applications as a bioactive food ingredient. A review. *Trends in Food Science &Technology*.

[84] Vosoghkia M., Ghareaghhag L. H., Ghavami M., Gharachorloo M., Delkosh B. (2011). Evaluation of oil content and fatty acid composition in seeds of different genotypes of safflower (Carthamus tinctorius L.). *International Journal of Agricultural Science and Research*.

[85] Rotunda A. M., Kolodney M. S. (2006). Mesotherapy and phosphatidylcholine injections: historical clarification and review. *Dermatologic Surgery*.

[86] Proctor V. A., Cunningham F. E. (1988). The chemistry of lysozyme and its use as a food preservative and a pharmaceutical. *Critical Reviews in Food Science & Nutrition*.

[87] Abdel Moneim E. S., Sherif M. S., Ahmed A. E., Mohanad A., Vajid N. V. (2018). Evaluation of antimicrobial activity of safflower (Carthamus tinctorius) and its synergistic effect with antibiotic. *EC Microbiology*.

[88] Jaberian H., Piri K., Nazari J. (2013). Phytochemical composition and in vitro antimicrobial and antioxidant activities of some medicinal plants. *Food Chemistry*.

[89] Kchaou W., Abbès F., Mansour R. B., Blecker C., Attia H., Besbes S. (2016). Phenolic profile, antibacterial and cytotoxic properties of second grade date extract from Tunisian cultivars (Phoenix dactylifera L.). *Food Chemistry*.

[90] Üstündağ Ö. G., Erşan S., Özcan E., Özan G., Kayra N., Ekinci F. Y. (2016). Black tea processing waste as a source of antioxidant and antimicrobial phenolic compounds. *European Food Research and Technology*.

